# Cyanophage MazG is a pyrophosphohydrolase but unable to hydrolyse magic spot nucleotides

**DOI:** 10.1111/1758-2229.12741

**Published:** 2019-03-20

**Authors:** Branko Rihtman, Sabine Bowman‐Grahl, Andrew Millard, Rebecca M. Corrigan, Martha R. J. Clokie, David J. Scanlan

**Affiliations:** ^1^ School of Life Sciences University of Warwick Coventry UK; ^2^ Department of Infection, Immunity and Inflammation University of Leicester Leicester UK; ^3^ Department of Molecular Biology & Biotechnology University of Sheffield Sheffield UK

## Abstract

Bacteriophage possess a variety of auxiliary metabolic genes of bacterial origin. These proteins enable them to maximize infection efficiency, subverting bacterial metabolic processes for the purpose of viral genome replication and synthesis of the next generation of virion progeny. Here, we examined the enzymatic activity of a cyanophage MazG protein – a putative pyrophosphohydrolase previously implicated in regulation of the stringent response via reducing levels of the central alarmone molecule (p)ppGpp. We demonstrate, however, that the purified viral MazG shows no binding or hydrolysis activity against (p)ppGpp. Instead, dGTP and dCTP appear to be the preferred substrates of this protein, consistent with a role preferentially hydrolysing deoxyribonucleotides from the high GC content host *Synechococcus* genome. This showcases a new example of the fine‐tuned nature of viral metabolic processes.

## Introduction

Cyanophage that infect the marine cyanobacterial genera *Synechococcus* and *Prochlorococcus* are widespread and abundant in oceanic systems (Suttle and Chan, 1994; Sullivan *et al.,* 2003; Baran *et al.,* 2018) where they play important ecosystem roles including releasing organic matter through cell lysis (Suttle, 2007), transferring genes horizontally between hosts (Zeidner *et al.,* 2005) and structuring host communities (Mühling *et al.,* 2005). Cyanophage can also influence ocean biogeochemistry by modifying host metabolism during the infection process, such as the shutdown of CO_2_ fixation whilst maintaining photosynthetic electron transport (Puxty *et al.,* 2016). This subversion of host metabolism is facilitated by the expression of cyanophage genes that appear to have a bacterial origin, so‐called auxiliary metabolic genes (AMGs) (Breitbart *et al.,* 2007). These include genes involved in photosynthesis (Mann *et al.,* 2003; Lindell *et al.,* 2005; Fridman *et al.,* 2017) and photoprotection (Lindell *et al.,* 2004; Millard *et al.,* 2004; Sullivan *et al.,* 2005; Roitman *et al.,* 2018), pigment biosynthesis (Dammeyer *et al.,* 2008), central carbon metabolism (Millard *et al.,* 2009; Thompson *et al.,* 2011), nucleotide biosynthesis (Enav *et al.,* 2014), phosphorus metabolism (Sullivan *et al.,* 2010; Zeng and Chisholm, 2012; Lin *et al.,* 2016) and other stress responses (Sullivan *et al.,* 2010; Crummett *et al.,* 2016).

Amongst the cyanophage AMGs MazG is a core gene in cyanomyoviruses (Millard *et al.,* 2009; Sullivan *et al.,* 2010) and of particular interest since it has been proposed to play a more general role in regulating host metabolism (Clokie and Mann, 2006; Clokie *et al.,* 2010). In *Escherichia coli,* MazG has been implicated in regulating programmed cell death by interfering with the function of the MazEF toxin‐antitoxin system, through lowering of cellular (p)ppGpp levels (Gross *et al.,* 2006). This latter molecule guanosine 3′,5′ bispyrophosphate, together with guanosine pentaphosphate also known as magic spot nucleotides, is a global regulator of gene expression in bacteria (Traxler *et al.,* 2008) synthesized by RelA under amino acid starvation. Since MazG can potentially regulate levels of (p)ppGpp in *E. coli,* a similar role has been proposed for the cyanophage encoded MazG (Clokie and Mann, 2006). This is pertinent given that picocyanobacterial hosts like *Synechococcus* and *Prochlorococcus* occupy oligotrophic conditions (see Scanlan *et al.,* 2009; Biller *et al.,* 2015) where nutrient starvation is likely and (p)ppGpp may be involved in adapting to this stressed state. By regulating (p)ppGpp levels the cyanophage encoded MazG may trick the host into mimicking a nutrient replete cellular state so that host cell physiology is optimized for macromolecular synthesis and hence cyanophage replication. The MazG protein belongs to the all‐nucleoside triphosphate pyrophosphohydrolase (NTP‐PPase, EC 3.6.1.8) superfamily that hydrolyzes *in vitro* all canonical nucleoside triphosphates into monophosphate derivatives and pyrophosphate (PPi) (Moroz *et al.,* 2005; Galperin *et al.,* 2006; Lu *et al.,* 2010). Here, we set out to purify the cyanophage S‐PM2 MazG protein as well as a *Synechococcus* host MazG to assess their activity and ability to hydrolyse (p)ppGpp, canonical and noncanonical nucleotides.

## Results

**Figure 1:**
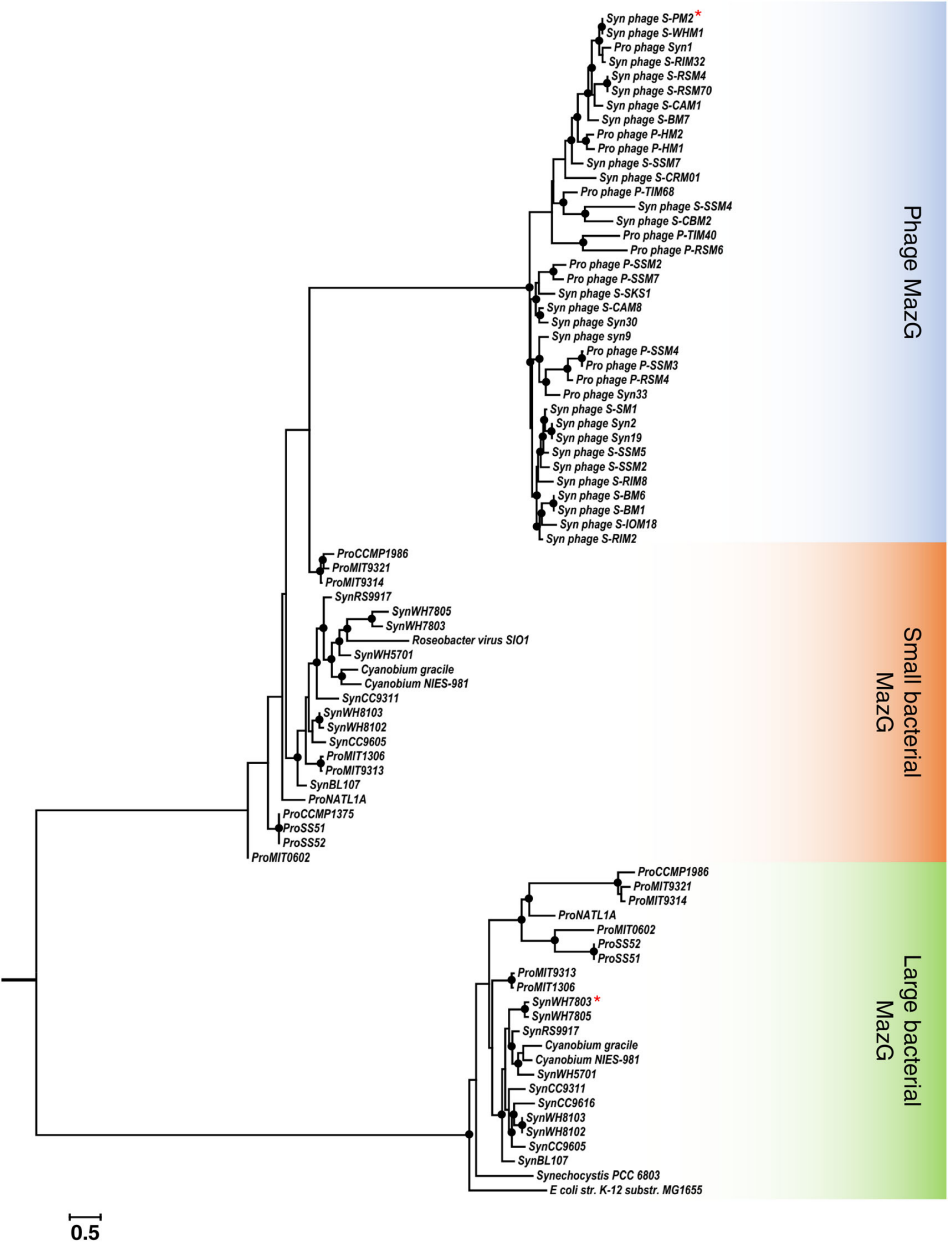
Maximum likelihood phylogenetic tree comprising 44 bacterial and 38 viral MazG sequences. The tree was generated using the LG + G4 substitution model, automatically chosen by the Iqtree script (Nguyen *et al.,* 2015), with ultrafast bootstrap (Minh *et al.,* 2013). Bootstrap values of >70% are shown as closed circles (of 1000 iterations). The scale bar represents 0.5 substitutions/amino acid position. Syn: *Synechococcus*; Pro: *Prochlorococcus*. The red asterisks indicate the *Synechococcus* and cyanophage proteins used here.

**Figure 2:**
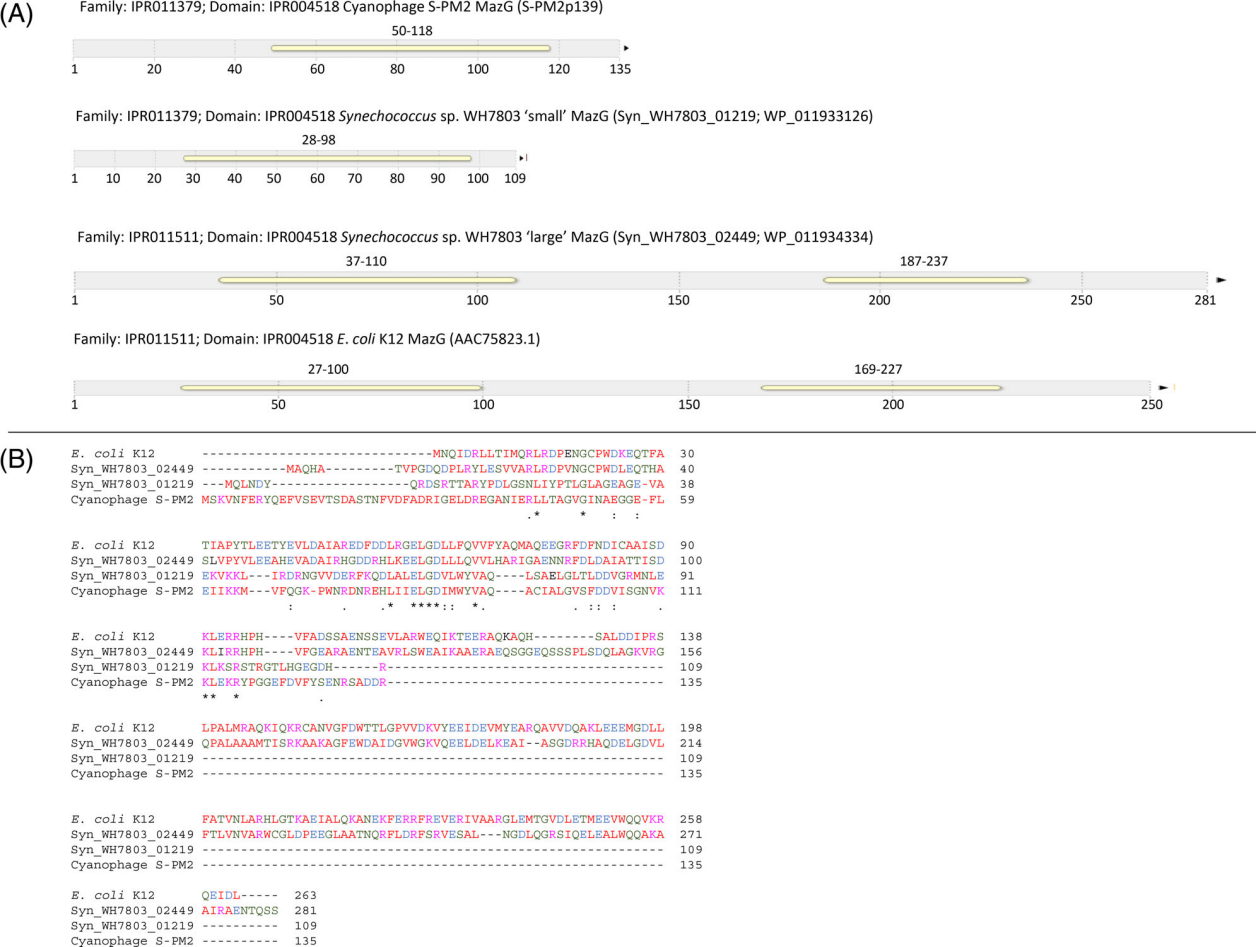
A. InterProScan5‐predicted (Jones *et al.,* 2014) pyrophosphatase catalytic domains in cyanophage S‐PM2 MazG, ‘small’ *Synechococcus* sp. WH7803 MazG (Syn_WH7803_01219), ‘large’ *Synechococcus* sp. WH7803 MazG (Syn_WH7803_02449) and *E. coli* MazG orthologues. Numbers above each domain represent the position of amino acids in each of the domains. B. ClustalW pairwise alignment of *E. coli*, ‘large’ *Synechococcus* sp. WH7803, ‘small’ *Synechococcus* sp. WH7803 and cyanophage S‐PM2 MazG orthologues.

**Figure 3:**
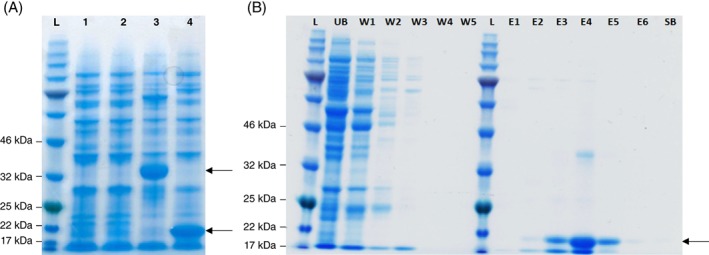
A. SDS‐PAGE analysis of *E. coli* whole cell lysates expressing *Synechococcus* sp. WH7803 ‘large’ MazG (lanes 1 and 3) and cyanophage S‐PM2 MazG proteins (lanes 2 and 4). L: Protein molecular weight marker ladder. Lanes 1 and 2 un‐induced, lanes 3 and 4 IPTG‐induced. Arrows indicate the positions of the overexpressed proteins. B. SDS‐PAGE analysis showing purification of the cyanophage S‐PM2 MazG protein from *E. coli*. L: Protein molecular weight marker ladder. UB: The unbound fraction (proteins that did not bind to the column). W1–W5: fractions washed off the column with binding buffer. E1–E6: Fractions eluted with increasing concentrations of imidazole (30 mM, 50 mM, 100 mM, 150 mM, 200 mM and 300 mM respectively). SB – stripping buffer. The arrow indicates the position of the over‐expressed cyanophage S‐PM2 MazG protein.

**Figure 4:**
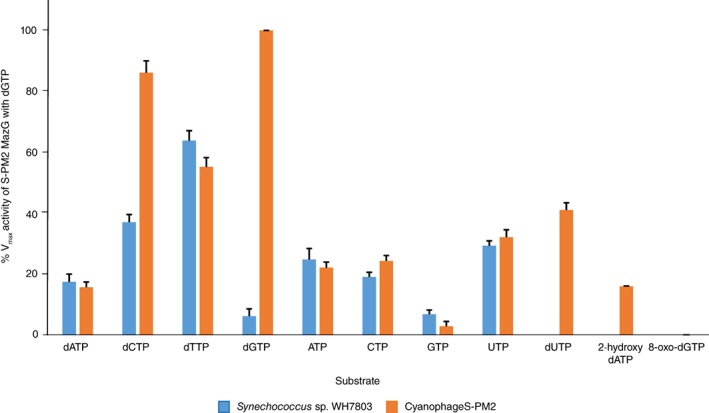
Relative maximal activity (V_max_) of the *Synechococcus* sp. WH7803 ‘large’ MazG and cyanophage S‐PM2 MazG proteins against a range of canonical and noncanonical nucleotide and deoxyribonucleotide substrates, normalized to the activity of the cyanophage S‐PM2 MazG using dGTP as a substrate. Error bars represent the standard error based on three replicate experiments.

Picocyanobacterial host and cyanophage MazG proteins are phylogenetically distinct (Fig. 1) and with an origin of the cyanophage MazG outside the cyanobacteria since the closest proposed homologue to date is a *Chloroflexus* protein (Bryan *et al.,* 2008; Sullivan *et al.,* 2010). Picocyanobacteria encode two genes annotated as MazG, a ‘large’ MazG version similar to that found in most bacteria, and a ‘small’ version which is similar in size to the cyanophage gene (Fig. 2). The ‘large’ MazG version has two predicted catalytic regions functionally annotated as MazG family domains (IPR004518) whilst the ‘small’ MazG and cyanophage proteins have only one (Fig. 2). In order to assess the hydrolytic activity of the host and cyanophage MazG proteins we cloned into *E. coli*, over‐expressed and purified the host *Synechococcus* sp. WH7803 MazG, using the ‘large’ MazG version (Syn_WH7803_02449) as a proxy for other host bacterial MazG proteins, and the cyanophage S‐PM2 MazG (Fig. 3; for experimental details see Supporting Information). The activity of the cyanophage and *Synechococcus* host MazG proteins was assessed using increasing concentrations of a range of nucleotide and deoxyribonucleotide substrates using 1 μg of the purified protein, and the amount of free phosphate resulting from enzyme activity measured using the PiPER pyrophosphate assay kit (ThermoFisher Scientific; see Supporting Information). This allowed determination of *K*_m_, *V*_max_ and *K*_cat_ values for each protein across a range of substrates (Table 1). *K*_m_ values of the *Synechococccus* sp. WH7803 ‘large’ MazG and cyanophage S‐PM2 MazG proteins were generally in the low mM range for a range of nucleotides and deoxyribonucleotides, similar to MazG *K*_m_ values reported from other bacteria for these substrates (Lu *et al.,* 2010). The measured *V*_max_ of the *Synechococcus* host MazG was highest when incubated with dTTP, whilst the viral MazG exhibited highest activity when incubated with the deoxyribonucleotides dGTP and dCTP (Fig. 4). In addition to these standard nucleotides, the viral MazG protein was also incubated with the ‘aberrant’ nucleotides dUTP, 2‐hydroxy‐dATP and 8‐oxo‐dGTP. dUTP is one of the most common of these mutagenic nucleotides, produced as a by‐product of thymine biosynthesis (Galperin *et al.,* 2006), whilst 2‐hydroxy‐dATP and 8‐oxo‐dGTP are mutagenic nucleotides produced as a result of intracellular oxidative stress (Kamiya and Kasai, 2000; Galperin *et al.,* 2006). Interestingly, the *V*_max_ values of the viral MazG when incubated with dUTP, 2‐hydroxy‐dATP and 8‐oxo‐dGTP were not significantly different to those of the canonical nucleotides (Table 1; Fig. 4), whilst the Km values for these substrates were higher (Table 1), suggesting that dGTP and dCTP are the preferred substrates of the cyanophage MazG protein.

**Table 1:** Kinetic parameters of enzymatic activity of Synechococcus WH7803 and cyanophage S‐PM2 MazG protein.

	*V*_max_ (nmol/μg/min)		*K*_m_ (mM)		*K*_cat_ (min^−1^)	
	*Synechococcus* sp. WH7803	Cyanophage S‐PM2	*Synechococcus* sp. WH7803	Cyanophage S‐PM2	*Synechococcus* sp. WH7803	Cyanophage S‐PM2
dATP	1.8 (±0.28)	1.62 (±0.19)	0.3 (±0.09)	1.2 (±0.21)	126.12 (±19.35)	62.97 (±7.44)
dCTP	3.81 (±0.36)	8.86 (±0.2)	0.14 (±0.03)	1.16 (±0.04)	267.68 (±25.02)	344.68 (±7.72)
dTTP	6.57 (±0.19)	5.68 (±0.2)	ND	1.23 (±0.06)	461.04 (±13.43)	221.00 (±7.78)
dGTP	0.64 (±0.25)	10.29 (±0.25)	0.85 (±0.07)	0.14 (±0.01)	45.16 (±17.6)	400.35 (±9.91)
ATP	2.55 (±0.35)	2.28 (±0.24)	0.63 (±0.23)	1.43 (±0.36)	179.27 (±24.4)	88.7 (±9.41)
CTP	1.96 (±0.14)	2.51 (±0.17)	1.2 (±0.21)	0.85 (±0.11)	137.81 (±9.81)	97.48 (±6.68)
GTP	0.7 (±0.13)	0.3 (±0.02)	0.26 (±0.02)	ND	49.46 (±9.19)	11.67 (±0.6)
UTP	3.02 (±0.2)	3.31 (±0.18)	1.33 (±0.3)	0.6 (±0.37)	221.07 (±6)	128.75 (±7.12)
dUTP	‐	4.22 (±0.34)	‐	3.24 (±1.55)	‐	296.42 (±23.72)
2‐hydroxy d‐ATP	‐	1.65 (±0.06)	‐	4.86 (±1.13)	‐	115.65 (±3.95)
8‐oxo‐dGTP	‐	ND	‐	ND	‐	ND

The values in brackets represent *SE* based on three replicates. ND – not detected; − not measured.

**Figure 5:**
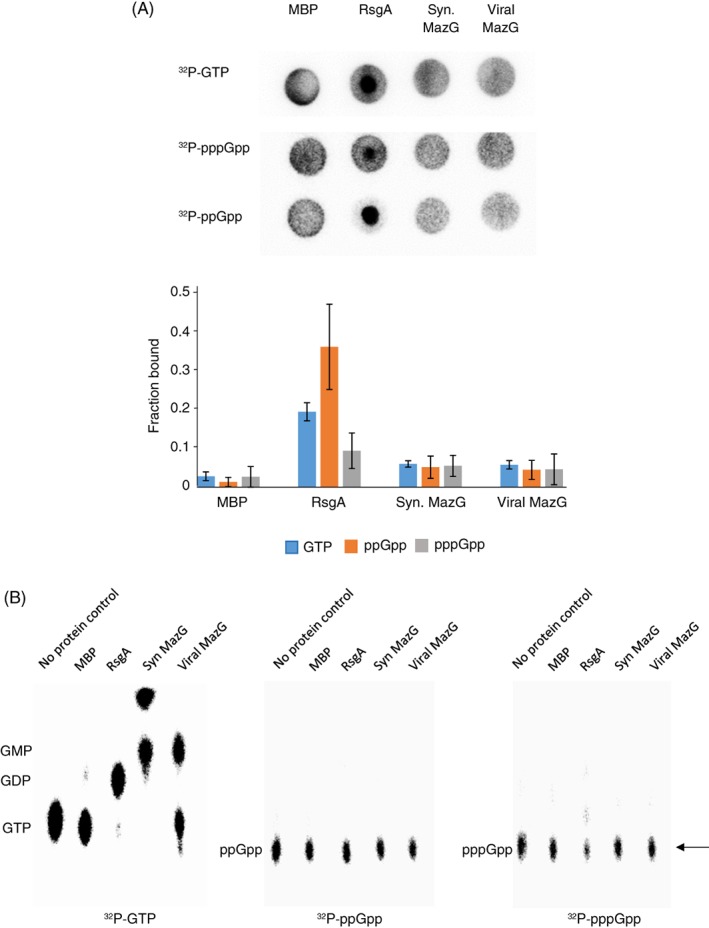
A. Upper panel: DRaCALA binding assays, using ^32^P‐labelled GTP, ppGpp and pppGpp incubated with purified *Synechococcus* sp. WH7803 ‘large’ MazG and cyanophage S‐PM2 MazG proteins. MBP – maltose binding protein, used as a negative control. RsgA *–*purified RsgA protein from *S. aureus*, used as a positive control. Syn MazG: *Synechococcus* sp. WH7803 ‘large’ MazG. Viral MazG: cyanophage S‐PM2 MazG. Lower panel: Bar chart representation of the fraction of substrate bound to each protein, as measured by densitometry. Syn. MazG: *Synechococcus* sp. WH7803 ‘large’ MazG. Viral MazG: cyanophage S‐PM2 MazG. Error bars represent the standard deviation of three experimental replicates. B. Hydrolysis assay using purified *Synechococcus* sp. WH7803 ‘large’ MazG (Syn MazG), cyanophage S‐PM2 MazG (Viral MazG), MBP and RsgA proteins with ^32^P‐labelled GTP, ppGpp and pppGpp. The arrow highlights the absence of hydrolysis of ^32^P‐labelled ppGpp and pppGpp substrates.

In order to directly assess whether the *Synechococcus* and cyanophage MazG proteins play a role in (p)ppGpp metabolism we performed both hydrolysis and DRaCALA binding assays (Corrigan *et al.,* 2016), using ^32^P‐labelled GTP, ppGpp and pppGpp. In both assays, neither the *Synechococcus* nor cyanophage MazG showed any binding or hydrolysis activity against (p)ppGpp (Fig. 5A), whilst hydrolysis activity was confirmed for both orthologues against ^32^P‐labelled GTP (Fig. 5B).

## Discussion

Although, the presence and identity of AMGs in bacteriophage genomes is widely appreciated (Millard *et al.,* 2009; Sullivan *et al.,* 2010; Crummett *et al*., 2016) the specific role of many of these genes has not been resolved. Here, we sought to elucidate the activity of the cyanophage MazG protein given its hypothesized role as a more general modulator of the host stringent response, and with previous data suggesting cyanophage can modulate intracellular levels of (p)ppGpp in infected freshwater cyanobacteria (Borbély *et al.,* 1980).

Our results showed, however, that neither the *Synechococcus* nor cyanophage MazG protein demonstrated detectable hydrolytic activity towards ppGpp or pppGpp (Fig. 5), suggesting these two proteins do not actively modulate the stringent response via direct hydrolysis of magic spot nucleotides. Nevertheless, we cannot rule out a role for these proteins in regulating the stringent response indirectly through hydrolysis of other nucleotide substrates, for example GTP. Whilst the role of the ‘small’ *Synechococcus* host MazG also requires clarification in this respect, it is potentially the predicted bifunctional *Synechococcus* sp. WH7803 SpoT orthologue (SynWH7803_2342) that serves the role of regulating alarmone levels during the stringent response in these organisms, a protein known to both synthesize and hydrolyse (p)ppGpp in other bacteria (see, e.g. Murray and Bremer, 1996; Hogg *et al.,* 2004). Interestingly, there were distinct differences in the hydrolytic activities of the *Synechococcus* host and cyanophage S‐PM2 MazG proteins towards other canonical and noncanonical nucleotides (Fig. 4 and Table 1) with much higher *V*_max_ values of the viral MazG towards dGTP and dCTP coupled with a much higher affinity of the viral MazG for dGTP compared to its host counterpart. Such different kinetic parameters mirror differences in %GC content between the cyanophage and *Synechococcus* host genomes, with the former possessing a GC content of 37.7% (Mann *et al.,* 2005) and the latter a GC content of 60.2% (Dufresne *et al.,* 2008). With this in mind, we suggest that the substrate specificity of the viral MazG allows it to preferentially hydrolyse dGTP and dCTP deoxyribonucleotides from the high GC content host *Synechococcus* genome allowing for their recycling and ultimately facilitating replication of the AT‐rich cyanophage genome. Whether such a mechanism is applicable to, or modified in, *Prochlorococcus* infecting cyanophage whose genomes generally possess a similar %GC content (Sullivan *et al.,* 2005; Limor‐Waisberg *et al.,* 2011) remains to be determined. Certainly, it is well known that following infection with cyanophage, the host genome is rapidly degraded (Doron *et al.,* 2016). Moreover, analysis of viral metagenomes has shown an enrichment of metabolic pathways involved in pyrimidine and purine metabolism as well as in DNA replication (Enav *et al.,* 2014), emphasizing the importance of these pathways during viral infection.

Our work with the viral MazG thus highlights that cyanophage genomes appear exquisitely suited to promote degradation of the host genome in order to reuse its building blocks to replicate the viral genome.

## Supporting information


**Appendix S1:** Supplementary Information
