# Supplementary material for: Cyanophage MazG is a pyrophosphohydrolase but unable to hydrolyse magic spot nucleotides
Source: Environ Microbiol Rep. 2019 Mar 20;11(3):448–55. doi: 10.1111/1758-2229.12741 (PMC6850273; doi:10.1111/1758-2229.12741)
Supplement: Supplementary file 1 — Appendix S1: Supplementary Information [file EMI4-11-448-s001.docx]

**Supplementary Information**

**Materials and Methods**

**Cloning of the *Synechococcus* host and cyanophage *mazG* genes**

The *Synechococcus* sp. WH7803 ‘large’ *mazG* (Syn_WH7803_02449; WP_011934334) and cyanophage S-PM2 *mazG* (S-PM2p139; YP_195171) genes were codon-optimized for over-expression of the respective proteins in *E. coli* using the GeneArt gene synthesis service (Thermofisher Scientific^TM^). Codon optimised sequences of the host and cyanophage *mazG* genes are shown in Supplementary Figures S1 and S2, respectively. The codon-optimised host and cyanophage *mazG* genes were then cloned into pET151d-TOPO^®^, allowing production of his-tagged proteins according to the manufacturer’s instructions (Thermofisher Scientific^TM^), transformed into *E. coli* BL21 Star™(DE3) and constructs checked by Sanger sequencing (GATC Biotech).

**Over-expression of** ***Synechococcus* host and cyanophage MazG proteins in *E*. *coli***

*E*. *coli* BL21 Star™(DE3) containing each over-expression construct was incubated overnight at 37ºC with shaking (300 rpm) in LB medium containing 50 µg ml^-1^ ampicillin, 1% (w/v) glucose (used to repress leaky expression of the lac promoter). This starter culture was used to inoculate 1L of MagicMedia *E*. *coli* Expression Medium (ThermoFisher ScientificTM) and incubated overnight at 30ºC with shaking (300 rpm). Cells were harvested by centrifugation, the cell pellet resuspended in binding buffer (20 mM Tris-HCl pH 8.0, 0.5 M NaCl, 10% (v/v) glycerol, 10 mM imidazole) and cells lysed by passage through a French Press (Aminco). Lysate was separated into soluble and insoluble fractions by centrifugation at 10,000 x g for 10 min at 4°C and the presence of over-expressed protein assessed using SDS-PAGE. Cyanophage S-PM2 MazG was found to be soluble and was purified as outlined below, whereas the *Synechococcus* sp. WH7803 MazG protein was found in the insoluble fraction and purified from inclusion bodies (see below).

**Purification of cyanophage S-PM2 MazG**

Protein extract containing the over-expressed cyanophage S-PM2 MazG protein was resuspended in binding buffer (20 mM Tris-HCl pH 8.0, 0.5 M NaCl, 10% (v/v) glycerol, 10 mM imidazole) and applied to a HisTrap HP column (GE Healthcare) i^++^ affinity column. The column was washed with 5 volumes of binding buffer (25 ml) and the protein eluted using a stepwise-increase in imidazole concentration (30-300 mM) in binding buffer (see Fig. 3B in the main text). Subsequently, the column was stripped with stripping buffer (500 mM NaCl, 50 mM EDTA, pH 8.0) and an aliquot of each fraction analysed by SDS-PAGE (see below).

**Purification of *Synechococcus* sp. WH7803 ‘large’ MazG**

The insoluble fraction (see above) was resuspended in 10ml g^-1^ 20 mM Tris-HCl pH 8; 150 mM NaCl, 5 mM MgCl_2_ and treated with 100 µg ml^-1^ DNAseI (New England Biolabs) for 15 min at 37ºC. Cell debris was pelleted by centrifugation at 20,000g, 15 min, 4°C. The pellet was denatured in 50 ml g^-1^ Buffer A (6M guanidine hydrochloride, 20 mM Tris-HCl pH 8; 500 mM NaCl, 10 mM imidazole). Protein was loaded onto a HisTrap HP (GE Healthcare) Ni^++^ affinity column and washed with 3 column volumes (CV) of Buffer A. S-PM2-His_6_-MazG was refolded in Buffer B (as Buffer A, but without guanidine hydrochloride) over 10 CV and finally gradually eluted in Buffer C (as Buffer B, but with 500 mM imidazole) over 5 CV. Fractions containing His_6_-MazG were pooled and dialysed against 20 mM Tris-HCl pH 8; 100 mM NaCl, 20% (v/v) glycerol overnight at 4°C. The protein was concentrated to ~1 μg μl^-1^ using an Amicon ultra centrifuge filter (Amicon, MWCO 3,000) and stored at -20°C.

**SDS-PAGE analysis**

Purified cyanophage S-PM2 MazG was observed as a polypeptide of ~19kDa via SDS-PAGE analysis (Figure 3B, lane E4), consistent with the predicted size of the cyanophage MazG protein. A larger ca 38 kDa fainter band was also observed in the purified fraction and mass spectrometry of this polypeptide using a Thermo Orbitrap Fusion mass spectrometer (Q-OT-qIT, Thermo Scientific) at the University of Warwick Proteomics facility also identified this as the cyanophage S-PM2 MazG protein, suggesting a small amount of dimerization of the protein.

**MazG activity assays**

Purified MazG activity was assessed against a range of substrates using a method previously described by Lu*, et al.*, (2010). Briefly, purified protein in Binding Buffer (20 mM Tris-HCl pH 8; 100 mM NaCl, 20% (v/v) glycerol) was incubated in triplicate with varying concentrations of different nucleotide and deoxyribonucleotide substrates at 37ºC for 10 minutes, followed by an inactivation step at 70ºC for 10 minutes. The inorganic phosphate released was then assessed using a PiPER pyrophosphate assay kit (ThermoFisher Scientific), per the manufacturer’s instructions. Fluororescence was measured using a ClarioStar plate reader (BMG-Labtech) with excitation and emission wavelengths of 550nm and 590nm, respectively.

The hydrolytic activity of purified cyanophage S-PM2 and *Synechococcus* sp. WH7803 MazG proteins against α-^32^P-labeled ppGpp and α-^32^P-labeled -pppGpp was performed using a hydrolysis assay and a DRaCALA binding assay as follows: For the DRaCALA binding assay 80.7 µM bacterial MazG, 101 µM cyanophage MazG, 330 µM MBP (negative control) and 142.6 µM *S. aureus* RsgA (positive control) were incubated in binding buffer (40 mM Tris pH7.5, 100 mM NaCl, 10 mM MgCl_2_) with 278 nM α-^32^P labelled nucleotides for 5 minutes at room temperature. 2.5 µl of the binding reaction was then spotted onto a nitrocellulose membrane, allowed to dry and exposed to IP for 5 minutes. The membranes were then imaged on a LA 7000 Typhoon PhosphorImager and the intensity and area of inner and outer spots estimated using densitometry. Hydrolysis assays were performed using identical amounts of protein and α-^32^P-labeled nucleotides and buffer. Binding reactions were incubated at 37°C for 1hr and the protein then precipitated out of solution using 1.2M final concentration formic acid. The reaction tubes were centrifuged for 5 min at 17,000xg and 1µl was spotted onto PEI cellulose F-TLC plates followed by separation in 1 M KH_2_PO_4_, pH 3.6 buffer. The radioactive spots were visualized using an LA 7000 Typhoon PhosphorImager, and images were quantified using ImageJ.

**References**

Lu, L.D., Sun, Q., Fan, X.Y., Zhong, Y., Yao, Y.F., & Zhao, G.P. (2010). Mycobacterial MazG is a novel NTP pyrophosphohydrolyase involved in oxidative stress response. *J Biol Chem* **285:** 28076-28085.

Supplementary Figure S1. Codon optimised sequence of the *Synechococcus* sp. WH7803 *mazG* gene before (WH7803) and after optimization (Opt.) for *E. coli* codon usage. The modified codons in the optimized sequence are underlined.

WH7803 ATGGCCCAGCACGCCACGGTTCCTGGTGATCAGGATCCCCTGCGCTACCTCGAAAGCGTG
 M A Q H A T V P G D Q D P L R Y L E S V
Opt. ATGGCACAGCATGCAACCGTTCCGGGTGATCAGGATCCGCTGCGTTATCTGGAAAGCGTT

WH7803 GTGGCACGCCTGCGGGATCCAGTGAACGGGTGCCCCTGGGACCTGGAGCAGACCCATGCC
 V A R L R D P V N G C P W D L E Q T H A
Opt. GTTGCACGTCTGCGTGATCCGGTTAATGGTTGTCCGTGGGATCTGGAACAGACCCATGCA

WH7803 TCCCTGGTGCCCTACGTCCTGGAGGAAGCCCATGAGGTGGCTGACGCCATCCGCCATGGC
 S L V P Y V L E E A H E V A D A I R H G
Opt. AGCCTGGTTCCGTATGTTCTGGAAGAAGCACACGAAGTTGCAGATGCAATTCGTCATGGT

WH7803 GACGACCGGCATCTGAAAGAAGAACTTGGCGATCTGCTGCTGCAGGTGGTGCTGCACGCC
 D D R H L K E E L G D L L L Q V V L H A
Opt. GATGATCGTCATCTGAAAGAAGAACTGGGCGATCTGCTGCTGCAGGTTGTTCTGCATGCA

WH7803 CGCATCGGCGCAGAAAACAACCGCTTTGATCTCGATGCCATCGCCACGACCATCAGCGAC
 R I G A E N N R F D L D A I A T T I S D
Opt. CGTATTGGTGCAGAAAATAATCGTTTTGATCTGGATGCAATTGCCACCACCATTAGCGAT

WH7803 AAACTGATCCGCCGCCATCCCCACGTGTTCGGCGAGGCCCGAGCGGAAAACACTGAGGCC
 K L I R R H P H V F G E A R A E N T E A
Opt. AAACTGATTCGTCGTCATCCGCATGTTTTTGGTGAAGCACGTGCCGAAAATACCGAAGCA

WH7803 GTGCGCTTGAGCTGGGAGGCCATCAAGGCCGCCGAACGGGCGGAACAATCGGGAGGGGAG
 V R L S W E A I K A A E R A E Q S G G E
Opt. GTTCGTCTGAGCTGGGAAGCAATTAAAGCAGCAGAACGTGCAGAACAGAGCGGTGGTGAA

WH7803 CAATCCTCCAGCCCGCTGAGCGATCAACTGGCCGGCAAGGTGCGAGGGCAACCGGCCCTG
 Q S S S P L S D Q L A G K V R G Q P A L
Opt. CAGAGCAGCAGTCCGCTGAGCGATCAGCTGGCAGGTAAAGTTCGTGGTCAGCCTGCACTG

WH7803 GCCGCTGCCATGACCATCTCGCGCAAGGCCGCCAAGGCCGGTTTCGAGTGGGATGCCATC
 A A A M T I S R K A A K A G F E W D A I
Opt. GCAGCAGCAATGACCATTAGCCGTAAAGCAGCCAAAGCAGGTTTTGAATGGGATGCCATT

WH7803 GACGGCGTGTGGGGGAAGGTGCAGGAGGAGCTCGATGAGCTCAAGGAGGCCATCGCCTCG
 D G V W G K V Q E E L D E L K E A I A S
Opt. GATGGTGTTTGGGGTAAAGTGCAAGAGGAACTGGATGAACTGAAAGAGGCCATTGCATCA

WH7803 GGGGACCGCCGCCATGCTCAAGATGAGCTCGGCGATGTGCTGTTCACCCTTGTGAATGTG
 G D R R H A Q D E L G D V L F T L V N V
Opt. GGCGATCGTCGCCATGCACAGGATGAGCTGGGTGATGTTCTGTTTACCCTGGTTAATGTT

WH7803 GCCCGCTGGTGCGGCCTTGATCCTGAGGAGGGGCTGGCCGCAACCAATCAGCGCTTCCTG
 A R W C G L D P E E G L A A T N Q R F L
Opt. GCCCGTTGGTGTGGTCTGGATCCGGAAGAAGGTCTGGCAGCAACCAATCAGCGTTTTCTG

WH7803 GATCGTTTCTCCCGGGTTGAGAGCGCCCTGAATGGAGATCTGCAAGGACGGAGCATCCAG
 D R F S R V E S A L N G D L Q G R S I Q
Opt. GATCGTTTTAGCCGTGTTGAAAGCGCACTGAATGGTGATCTGCAGGGTCGTAGCATTCAA

WH7803 GAGCTCGAGGCTCTGTGGCAGCAAGCGAAGGCTGCGATCCGCGCTGAAAACACCCAGTCG
 E L E A L W Q Q A K A A I R A E N T Q S
Opt. GAACTGGAAGCACTGTGGCAGCAGGCAAAAGCAGCAATTCGTGCGGAAAACACCCAGAGC

WH7803 TCTTGA
 S *
Opt. AGCTAG

Supplementary Figure S2. Codon optimised sequence of the cyanophage S-PM2 *mazG* gene (S-PM2p139; YP_195171) before (S-PM2) and after optimization (Opt.) for *E. coli* codon usage. The modified codons in the optimized sequence are underlined.

S-PM2 ATGTCTAAAGTAAACTTTGAACGCTATCAAGAATTTGTGTCGGAAGTTACTTCCGATGCT M S K V N F E R Y Q E F V S E V T S D A
Opt. ATGAGCAAAGTGAACTTTGAGCGCTATCAAGAATTTGTTAGCGAAGTTACCAGTGATGCC

S-PM2 TCTACAAACTTCGTTGACTTCGCTGATCGTATTGGCGAGTTGGATCGTGAAGGTGCCAAT
 S T N F V D F A D R I G E L D R E G A N
Opt. AGCACCAATTTTGTTGATTTTGCAGATCGTATTGGTGAACTGGATCGTGAAGGTGCAAAT

S-PM2 ATTGAACGACTTCTTACTGCTGGTGTTGGCATCAATGCTGAGGGTGGTGAGTTTCTTGAG I E R L L T A G V G I N A E G G E F L E
Opt. ATTGAACGTCTGCTGACCGCAGGCGTTGGTATTAATGCCGAAGGTGGTGAATTTCTGGAA

S-PM2 ATCATTAAGAAGATGGTATTCCAAGGTAAGCCTTGGAACCGCGATAATCGAGAACATCTT
 I I K K M V F Q G K P W N R D N R E H L
Opt. ATCATCAAAAAAATGGTGTTCCAGGGTAAACCGTGGAATCGTGATAATCGTGAACATCTG

S-PM2 ATTATTGAGTTGGGTGACATTATGTGGTATGTGGCACAAGCATGTATTGCGCTAGGTGTT
 I I E L G D I M W Y V A Q A C I A L G V
Opt. ATTATTGAGCTGGGCGATATTATGTGGTATGTTGCACAGGCATGTATTGCACTGGGTGTT

S-PM2 TCTTTTGATGATGTCATTTCTGGCAACGTCAAGAAACTTGAAAAACGTTATCCAGGAGGA
 S F D D V I S G N V K K L E K R Y P G G
Opt. AGCTTTGATGATGTGATTAGCGGCAATGTGAAAAAACTGGAAAAACGTTATCCGGGTGGC

S-PM2 GAATTTGATGTCTTCTATTCCGAAAATAGATCAGCAGACGACCGATAA
 E F D V F Y S E N R S A D D R *
Opt. GAATTTGATGTGTTTTATAGCGAAAATCGTAGCGCAGATGATCGCTAG
